# *N*-(4,5-Diaza-9*H*-fluoren-9-yl­idene)-4-meth­oxy­aniline

**DOI:** 10.1107/S1600536810023275

**Published:** 2010-06-26

**Authors:** Hui Cang, Jin-Fa Bian, Si-Qing Wang, Jin-Tang Wang

**Affiliations:** a Chemical and Biological Engineering College, Yancheng Institute of Technology, Yancheng 224051, People’s Republic of China; b Department of Chemical Engineering, Nanjing College of Chemical Technology, Nanjing 210048, People’s Republic of China; c Department of Applied Chemistry, College of Science, Nanjing University of Technology, Nanjing 210009, People’s Republic of China

## Abstract

In the title compound, C_18_H_13_N_3_O, the diaza­fluorene ring system is almost coplanar (r.m.s. deviation = 0.0640 Å) and subtends an angle of 61.5 (4)° with the plane of the meth­oxy-substituted benzene ring. In the crystal structure, pairs of C—H⋯O hydrogen bonds link mol­ecules into centrosymmetric dimers parallel to the *ab* plane. Mol­ecules are also stacked in an obverse fashion along the *c* axis by a variety of π–π inter­actions with centroid–centroid distances in the range 3.557 (2)–3.921 (2) Å.

## Related literature

For the use of the title compound in the synthesis of complexes with inter­esting photochemical properties and for the synthesis, see: Wang & Rillema (1997 ▶). For reference bond-length data, see: Allen *et al.* (1987 ▶).
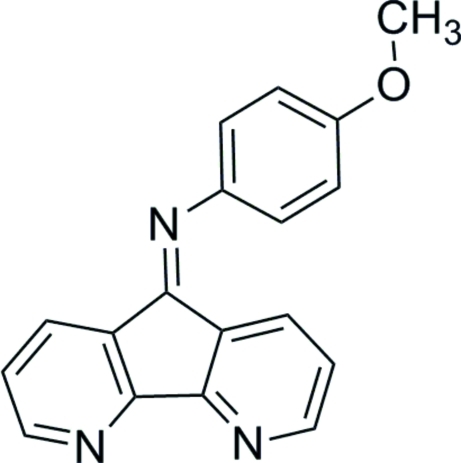


## Experimental

### 

#### Crystal data

C_18_H_13_N_3_O
*M* *_r_* = 287.31
Monoclinic, 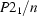

*a* = 8.3070 (17) Å
*b* = 12.839 (3) Å
*c* = 13.233 (3) Å
β = 97.12 (3)°
*V* = 1400.5 (5) Å^3^
*Z* = 4
Mo *K*α radiation
μ = 0.09 mm^−1^
*T* = 298 K
0.30 × 0.10 × 0.05 mm

#### Data collection

Enraf–Nonius CAD-4 diffractometer
Absorption correction: ψ scan (North *et al.*, 1968 ▶) *T*_min_ = 0.974, *T*_max_ = 0.996
2678 measured reflections
2498 independent reflections
1599 reflections with *I* > 2σ(*I*)
*R*_int_ = 0.025
3 standard reflections every 200 reflections  intensity decay: none

#### Refinement

*R*[*F* ^2^ > 2σ(*F* ^2^)] = 0.081
*wR*(*F* ^2^) = 0.212
*S* = 1.03
2498 reflections
193 parameters
48 restraints
H-atom parameters constrained
Δρ_max_ = 0.37 e Å^−3^
Δρ_min_ = −0.40 e Å^−3^

### 

Data collection: *CAD-4 Software* (Enraf-Nonius, 1985 ▶); cell refinement: *CAD-4 Software*; data reduction: *XCAD4* (Harms & Wocadlo, 1995 ▶); program(s) used to solve structure: *SHELXS97* (Sheldrick, 2008 ▶); program(s) used to refine structure: *SHELXL97* (Sheldrick, 2008 ▶); molecular graphics: *SHELXTL* (Sheldrick, 2008 ▶); software used to prepare material for publication: *SHELXTL*.

## Supplementary Material

Crystal structure: contains datablocks I, global. DOI: 10.1107/S1600536810023275/sj5020sup1.cif

Structure factors: contains datablocks I. DOI: 10.1107/S1600536810023275/sj5020Isup2.hkl

Additional supplementary materials:  crystallographic information; 3D view; checkCIF report

## Figures and Tables

**Table 1:** Hydrogen-bond geometry (Å, °)

*D*—H⋯*A*	*D*—H	H⋯*A*	*D*⋯*A*	*D*—H⋯*A*
C12—H12*A*⋯O^i^	0.93	2.42	3.337 (6)	169

Symmetry code: (i) 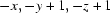.

**Fig. 1.**
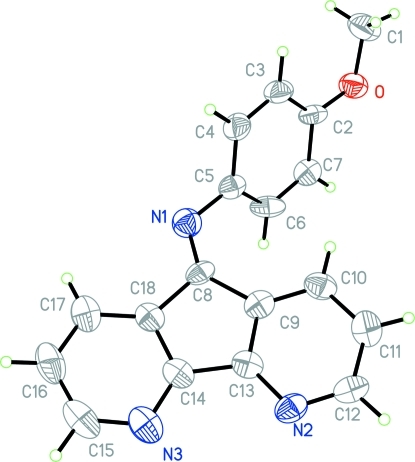
The structure or (I), with the atom-numbering scheme. Displacement ellipsoids are drawn at the 50% probability level.

**Fig. 2.**
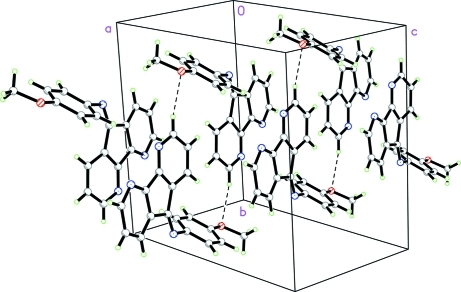
A packing diagram for (I). Hydrogen bonds are drawn as dashed lines.

## supplementary crystallographic information

### Comment

*N*-(5*H*-cyclopenta[1,2 - b:5,4 - b']dipyridin-5-ylidene)-4-methoxyaniline and its derivatives are an important class of ligands, being utilized to synthesize complexes with interesting photochemical properties (Wang & Rillema, 1997). Here we report the crystal structure of the title compound, (I).

The molecular structure of (I) is shown in Fig. 1. The bond lengths and angles are within normal ranges (Allen *et al.*, 1987). The diazafluorene rings are almost coplanar with an r.m.s. deviation 0.0640 Å and this plane is inclined to the plane of the C2···C7 benzene ring by 61.5 (4)°.

In the crystal structure C—H···O hydrogen bonds link molecules into centrosymmetric dimers parallel to the *ab* plane, Table 1. An extensive system of π–π contacts stacks molecules in an obverse fashion down the *c* axis, Fig. 2, with *Cg*1···*Cg*1 = 3.921 (2) Å, *Cg*2···*Cg*2 = 3.921 (2) Å and *Cg*1···*Cg*2 = 3.557 (2) Å. Symmetry operations 1/2-*X*, 1/2+Y, 1/2-*Z*; 3-*X*, –Y, –*Z*; *Cg*1 and *Cg*2 are the centroids of the C10,C9,C13,N2,C12,C11 and C18,C8,C9,C13,C14 rings, respectively.

### Experimental

The title compound was synthesized by a method reported in literature (Wang & Rillema, 1997). Crystals were obtained by dissolving the compound (2.0 g, 6.96 mmol) in ethyl acetate(50 ml), and evaporating the solvent slowly at room temperature for about 7 d.

### Refinement

All H-atoms were positioned geometrically and refined using a riding model with d(C-H) = 0.93Å,  U_iso_=1.2U_eq_ (C) for aromatic 0.96Å,  U_iso_ = 1.5U_eq_ (C) for CH_3_ atoms

### Crystal data

**Table:**

C_18_H_13_N_3_O	*F*(000) = 600
*M**_r_* = 287.31	*D*_x_ = 1.363 Mg m^−^^3^
Monoclinic, *P*2_1_/*n*	Mo *K*α radiation, λ = 0.71073 Å
Hall symbol: -P 2yn	Cell parameters from 25 reflections
*a* = 8.3070 (17) Å	θ = 9–12°
*b* = 12.839 (3) Å	µ = 0.09 mm^−^^1^
*c* = 13.233 (3) Å	*T* = 298 K
β = 97.12 (3)°	Block, colourless
*V* = 1400.5 (5) Å^3^	0.30 × 0.10 × 0.05 mm
*Z* = 4	

### Data collection

**Table:**

Enraf–Nonius CAD-4 diffractometer	1599 reflections with *I* > 2σ(*I*)
Radiation source: fine-focus sealed tube	*R*_int_ = 0.025
graphite	θ_max_ = 25.1°, θ_min_ = 2.2°
ω/2θ scans	*h* = −9→9
Absorption correction: ψ scan (North *et al.*, 1968)	*k* = 0→15
*T*_min_ = 0.974, *T*_max_ = 0.996	*l* = 0→15
2678 measured reflections	3 standard reflections every 200 reflections
2498 independent reflections	intensity decay: none

### Refinement

**Table:**

Refinement on *F*^2^	Primary atom site location: structure-invariant direct methods
Least-squares matrix: full	Secondary atom site location: difference Fourier map
*R*[*F*^2^ > 2σ(*F*^2^)] = 0.081	Hydrogen site location: inferred from neighbouring sites
*wR*(*F*^2^) = 0.212	H-atom parameters constrained
*S* = 1.02	*w* = 1/[σ^2^(*F*_o_^2^) + (0.050*P*)^2^ + 5.*P*] where *P* = (*F*_o_^2^ + 2*F*_c_^2^)/3
2498 reflections	(Δ/σ)_max_ = 0.001
193 parameters	Δρ_max_ = 0.37 e Å^−^^3^
48 restraints	Δρ_min_ = −0.40 e Å^−^^3^

### Special details

**Table:**

Geometry. All e.s.d.'s (except the e.s.d. in the dihedral angle between two l.s. planes) are estimated using the full covariance matrix. The cell e.s.d.'s are taken into account individually in the estimation of e.s.d.'s in distances, angles and torsion angles; correlations between e.s.d.'s in cell parameters are only used when they are defined by crystal symmetry. An approximate (isotropic) treatment of cell e.s.d.'s is used for estimating e.s.d.'s involving l.s. planes.
Refinement. Refinement of *F*^2^ against ALL reflections. The weighted *R*-factor *wR* and goodness of fit *S* are based on *F*^2^, conventional *R*-factors *R* are based on *F*, with *F* set to zero for negative *F*^2^. The threshold expression of *F*^2^ > σ(*F*^2^) is used only for calculating *R*-factors(gt) *etc*. and is not relevant to the choice of reflections for refinement. *R*-factors based on *F*^2^ are statistically about twice as large as those based on *F*, and *R*- factors based on ALL data will be even larger.

### Fractional atomic coordinates and isotropic or equivalent isotropic displacement parameters (Å^2^)

**Table:**

	*x*	*y*	*z*	*U*_iso_*/*U*_eq_	
O	0.1797 (4)	0.1191 (2)	0.5460 (2)	0.0455 (8)	
N1	−0.4360 (4)	0.2396 (3)	0.3622 (3)	0.045	
C1	0.2903 (6)	0.0674 (4)	0.4883 (4)	0.0580 (14)	
H1B	0.3927	0.0584	0.5298	0.087*	
H1C	0.3054	0.1085	0.4296	0.087*	
H1D	0.2473	0.0005	0.4668	0.087*	
N2	−0.4715 (5)	0.6162 (3)	0.3850 (3)	0.0417 (9)	
C2	0.0291 (5)	0.1441 (3)	0.4969 (3)	0.0354 (10)	
C3	−0.0260 (5)	0.1213 (3)	0.3959 (3)	0.0426 (11)	
H3A	0.0405	0.0853	0.3563	0.051*	
N3	−0.7987 (5)	0.5270 (3)	0.2939 (3)	0.0498 (11)	
C4	−0.1786 (5)	0.1517 (4)	0.3544 (3)	0.0425 (11)	
H4A	−0.2162	0.1317	0.2881	0.051*	
C5	−0.2786 (5)	0.2115 (3)	0.4081 (3)	0.0337 (10)	
C6	−0.2233 (6)	0.2327 (4)	0.5093 (3)	0.0448 (12)	
H6A	−0.2908	0.2678	0.5489	0.054*	
C7	−0.0708 (5)	0.2028 (3)	0.5526 (3)	0.0392 (10)	
H7A	−0.0342	0.2218	0.6193	0.047*	
C8	−0.4781 (5)	0.3355 (3)	0.3556 (3)	0.0325 (9)	
C9	−0.3939 (5)	0.4347 (3)	0.3850 (3)	0.0353 (10)	
C10	−0.2335 (5)	0.4605 (4)	0.4154 (3)	0.0429 (11)	
H10A	−0.1533	0.4096	0.4233	0.051*	
C11	−0.1965 (6)	0.5636 (4)	0.4337 (4)	0.0490 (12)	
H11A	−0.0902	0.5835	0.4554	0.059*	
C12	−0.3178 (6)	0.6378 (4)	0.4197 (3)	0.0433 (11)	
H12A	−0.2906	0.7066	0.4355	0.052*	
C13	−0.5040 (5)	0.5164 (3)	0.3679 (3)	0.0346 (10)	
C14	−0.6635 (5)	0.4748 (4)	0.3245 (3)	0.0363 (10)	
C15	−0.9247 (6)	0.4701 (5)	0.2532 (4)	0.0583 (15)	
H15A	−1.0208	0.5047	0.2307	0.070*	
C16	−0.9214 (6)	0.3612 (5)	0.2422 (4)	0.0564 (14)	
H16A	−1.0131	0.3252	0.2137	0.068*	
C17	−0.7832 (5)	0.3104 (4)	0.2738 (3)	0.0467 (12)	
H17A	−0.7777	0.2384	0.2674	0.056*	
C18	−0.6499 (5)	0.3656 (4)	0.3156 (3)	0.0365 (10)	

### Atomic displacement parameters (Å^2^)

**Table:**

	*U*^11^	*U*^22^	*U*^33^	*U*^12^	*U*^13^	*U*^23^
O	0.0423 (18)	0.0440 (19)	0.050 (2)	0.0081 (14)	0.0058 (14)	−0.0014 (15)
N1	0.045	0.045	0.045	0.000	0.006	0.000
C1	0.049 (3)	0.057 (3)	0.073 (4)	0.006 (2)	0.027 (3)	0.003 (3)
N2	0.053 (2)	0.035 (2)	0.038 (2)	−0.0018 (17)	0.0143 (17)	−0.0024 (17)
C2	0.047 (3)	0.023 (2)	0.039 (2)	0.0055 (18)	0.0183 (19)	−0.0003 (18)
C3	0.048 (3)	0.041 (3)	0.043 (3)	0.000 (2)	0.018 (2)	−0.011 (2)
N3	0.041 (2)	0.069 (3)	0.041 (2)	0.002 (2)	0.0099 (18)	0.011 (2)
C4	0.045 (3)	0.049 (3)	0.035 (2)	−0.006 (2)	0.010 (2)	−0.012 (2)
C5	0.042 (2)	0.026 (2)	0.035 (2)	−0.0025 (17)	0.0137 (18)	0.0017 (18)
C6	0.060 (3)	0.044 (3)	0.035 (3)	0.015 (2)	0.023 (2)	0.000 (2)
C7	0.048 (3)	0.040 (3)	0.031 (2)	−0.002 (2)	0.0088 (19)	0.002 (2)
C8	0.046 (2)	0.028 (2)	0.026 (2)	−0.0012 (18)	0.0140 (18)	−0.0042 (17)
C9	0.048 (2)	0.039 (2)	0.021 (2)	−0.0006 (18)	0.0102 (17)	0.0019 (18)
C10	0.042 (2)	0.041 (2)	0.046 (3)	0.0099 (19)	0.008 (2)	0.009 (2)
C11	0.046 (3)	0.055 (3)	0.046 (3)	−0.005 (2)	0.001 (2)	0.011 (2)
C12	0.059 (3)	0.034 (2)	0.037 (2)	0.007 (2)	0.009 (2)	−0.001 (2)
C13	0.039 (2)	0.041 (2)	0.026 (2)	0.0035 (18)	0.0123 (17)	0.0068 (18)
C14	0.036 (2)	0.048 (3)	0.027 (2)	0.0026 (19)	0.0131 (18)	0.008 (2)
C15	0.036 (3)	0.088 (4)	0.052 (3)	0.010 (3)	0.008 (2)	0.015 (3)
C16	0.033 (3)	0.087 (4)	0.051 (3)	−0.014 (3)	0.012 (2)	0.005 (3)
C17	0.043 (3)	0.059 (3)	0.039 (3)	−0.008 (2)	0.007 (2)	0.009 (2)
C18	0.036 (2)	0.051 (3)	0.024 (2)	0.003 (2)	0.0083 (17)	0.000 (2)

### Geometric parameters (Å, °)

**Table:**

O—C2	1.375 (5)	C6—H6A	0.9300
O—C1	1.429 (5)	C7—H7A	0.9300
N1—C8	1.280 (5)	C8—C9	1.482 (6)
N1—C5	1.418 (5)	C8—C18	1.510 (6)
C1—H1B	0.9600	C9—C10	1.383 (6)
C1—H1C	0.9600	C9—C13	1.392 (6)
C1—H1D	0.9600	C10—C11	1.375 (7)
N2—C13	1.323 (5)	C10—H10A	0.9300
N2—C12	1.331 (6)	C11—C12	1.382 (6)
C2—C3	1.389 (6)	C11—H11A	0.9300
C2—C7	1.397 (6)	C12—H12A	0.9300
C3—C4	1.374 (6)	C13—C14	1.477 (6)
C3—H3A	0.9300	C14—C18	1.412 (6)
N3—C14	1.328 (5)	C15—C16	1.407 (8)
N3—C15	1.333 (6)	C15—H15A	0.9300
C4—C5	1.389 (6)	C16—C17	1.341 (7)
C4—H4A	0.9300	C16—H16A	0.9300
C5—C6	1.388 (6)	C17—C18	1.372 (6)
C6—C7	1.378 (6)	C17—H17A	0.9300
			
C2—O—C1	117.6 (4)	C10—C9—C13	117.2 (4)
C8—N1—C5	120.2 (4)	C10—C9—C8	133.5 (4)
O—C1—H1B	109.5	C13—C9—C8	109.0 (4)
O—C1—H1C	109.5	C11—C10—C9	117.9 (4)
H1B—C1—H1C	109.5	C11—C10—H10A	121.1
O—C1—H1D	109.5	C9—C10—H10A	121.1
H1B—C1—H1D	109.5	C10—C11—C12	119.8 (4)
H1C—C1—H1D	109.5	C10—C11—H11A	120.1
C13—N2—C12	115.3 (4)	C12—C11—H11A	120.1
O—C2—C3	125.3 (4)	N2—C12—C11	123.7 (4)
O—C2—C7	116.2 (4)	N2—C12—H12A	118.1
C3—C2—C7	118.4 (4)	C11—C12—H12A	118.1
C4—C3—C2	120.1 (4)	N2—C13—C9	125.8 (4)
C4—C3—H3A	119.9	N2—C13—C14	124.9 (4)
C2—C3—H3A	119.9	C9—C13—C14	109.3 (4)
C14—N3—C15	116.0 (5)	N3—C14—C18	123.3 (4)
C3—C4—C5	122.2 (4)	N3—C14—C13	128.3 (4)
C3—C4—H4A	118.9	C18—C14—C13	108.4 (4)
C5—C4—H4A	118.9	N3—C15—C16	124.2 (5)
C6—C5—C4	117.1 (4)	N3—C15—H15A	117.9
C6—C5—N1	122.7 (4)	C16—C15—H15A	117.9
C4—C5—N1	120.0 (4)	C17—C16—C15	118.5 (5)
C7—C6—C5	121.6 (4)	C17—C16—H16A	120.7
C7—C6—H6A	119.2	C15—C16—H16A	120.7
C5—C6—H6A	119.2	C16—C17—C18	119.4 (5)
C6—C7—C2	120.4 (4)	C16—C17—H17A	120.3
C6—C7—H7A	119.8	C18—C17—H17A	120.3
C2—C7—H7A	119.8	C17—C18—C14	118.5 (4)
N1—C8—C9	133.8 (4)	C17—C18—C8	133.6 (4)
N1—C8—C18	120.6 (4)	C14—C18—C8	107.8 (4)
C9—C8—C18	105.5 (3)		
			
C1—O—C2—C3	1.4 (6)	C12—N2—C13—C9	1.5 (6)
C1—O—C2—C7	−174.4 (4)	C12—N2—C13—C14	−176.8 (4)
O—C2—C3—C4	−179.0 (4)	C10—C9—C13—N2	−5.1 (6)
C7—C2—C3—C4	−3.2 (6)	C8—C9—C13—N2	−179.8 (4)
C2—C3—C4—C5	4.4 (7)	C10—C9—C13—C14	173.3 (4)
C3—C4—C5—C6	−5.1 (7)	C8—C9—C13—C14	−1.3 (4)
C3—C4—C5—N1	−179.1 (4)	C15—N3—C14—C18	0.1 (6)
C8—N1—C5—C6	62.1 (6)	C15—N3—C14—C13	177.1 (4)
C8—N1—C5—C4	−124.2 (5)	N2—C13—C14—N3	1.3 (7)
C4—C5—C6—C7	4.9 (6)	C9—C13—C14—N3	−177.2 (4)
N1—C5—C6—C7	178.8 (4)	N2—C13—C14—C18	178.7 (4)
C5—C6—C7—C2	−4.1 (7)	C9—C13—C14—C18	0.2 (5)
O—C2—C7—C6	179.2 (4)	C14—N3—C15—C16	0.4 (7)
C3—C2—C7—C6	3.1 (6)	N3—C15—C16—C17	−0.4 (8)
C5—N1—C8—C9	1.7 (7)	C15—C16—C17—C18	0.0 (7)
C5—N1—C8—C18	−174.5 (3)	C16—C17—C18—C14	0.4 (6)
N1—C8—C9—C10	11.8 (8)	C16—C17—C18—C8	−178.2 (4)
C18—C8—C9—C10	−171.6 (4)	N3—C14—C18—C17	−0.5 (6)
N1—C8—C9—C13	−174.8 (5)	C13—C14—C18—C17	−178.0 (4)
C18—C8—C9—C13	1.8 (4)	N3—C14—C18—C8	178.5 (4)
C13—C9—C10—C11	4.6 (6)	C13—C14—C18—C8	0.9 (4)
C8—C9—C10—C11	177.6 (4)	N1—C8—C18—C17	−5.8 (7)
C9—C10—C11—C12	−1.1 (7)	C9—C8—C18—C17	177.0 (4)
C13—N2—C12—C11	2.6 (6)	N1—C8—C18—C14	175.5 (4)
C10—C11—C12—N2	−2.8 (7)	C9—C8—C18—C14	−1.6 (4)

### Hydrogen-bond geometry (Å, °)

**Table:**

*D*—H···*A*	*D*—H	H···*A*	*D*···*A*	*D*—H···*A*
C12—H12A···O^i^	0.93	2.42	3.337 (6)	169

Symmetry codes: (i) −*x*, −*y*+1, −*z*+1.
